# Bibliography

**Published:** 1909-10-15

**Authors:** 


### BIBLIOGRAPHY.

A MANUAL OF CHEMISTRY. A Guide to Lectures and Laboratory Work for Beginners in Chemistry. A Textbook specially adapted for students of Medicine, Pharmacy and Dentistry. By W. SIMON, Ph. D., M.D., Professor of Chemistry in the college of Physicians and Surgeons, Baltimore, and in the Baltimore College of Dental Surgery; Emeritus Professor in the Maryland College of Pharmacy; and DANIEL BASE, Ph. D., Professor of Chemistry in the Maryland College of Pharmacy. New (9th) edition, enlarged and thoroughly revised. Octavo, 716 pages, with 78 engravings and 9 colored plates, illustrating 64 of the most important chemical tests. Cloth, $3.00, *net.* Lea & Febiger, Philadelphia and New York, 1909.

Few text Books on any subject has met with such great favor in dental colleges as Simon's manual of Chemistry. This new edition, we predict, will command the continued confidence of teachers and such practitioners as are minded to keep their chemistry up to date. This manual will appeal to many because of its clearness of statement and its comprehensiveness. It treats of every phase of the science of dentistry, not of course with the completeness of the larger works on the separate branches. For the student of medicine and dentistry and as an aid to the study of the science in college it is remarkable for the very terse and satisfactory treatment given to the wide range of General, Organic, Analytic and Physiologic Chemistry. If every dental student could master the text of this work he would have an ideal working knowledge of chemistry that would meet al his practical requirements. It is this complete and compact treatment of the subject that has given this work its popularity. The present edition has had much new work put on it by Professor Base and he has because of his practical knowledge of the technical application to pharmacy made it especially valuable to pharmacy students. We regret that practitioners take so little interest in the fundamental sciences but we believe that no practitioner of dentistry could spend the small price of this book to greater advantage, and especially should he review his chemistry and bring it up to date. It is an ideal book for students, and practitioners who care for this subject will find it contains a good statement of modern Chemical Science. The book is beautifully made and a credit to the publishers.

THE PRINCIPLES OF BACTERIOLOGY. A Practical Manual for Students and Physicians. By A. C. ABBOTT, M.D., Professor of Hygiene, University of Pennsylvania. New (8th) edition, thoroughly revised. 12mo, 631 pages, with 100 illustrations, 26 in colors. Cloth, $2.75, *net.* Lea & Febiger, Philadelphia and New York, 1909.

This new edition gives evidence that a vast amount of work has been done in the science of Bacteriology and that in a quarter of a century this whole subject has developed till now it occupies one of the most important places in the curriculum of every medical and dental college. The unfortunate fact with dentistry is that there is so little demand for specialized books on this subject that we are compelled to get our knowledge and keep in touch with the science almost entirely by keeping in touch with the work of medical investigators and teachers instead of dental instructors. The splendid text book of Dr. Miller never sold beyond the first edition. It was applied and experimental bacteriology, consequently we must keep in touch with bacteriological advances from the text books of medical scientists. No text book on this subject has been better received than Abbott's. The reasons are clearly because of its clear adequate treatment of the subject. It is both a students manual and a scientific compendium of the important advancement in the science of Bacteriology. This new edition seems to have incorporated all that is new and eliminated some things that have not held their ground against the more recent research. The historic part of the book of course has changed but little, likewise the technical details. The modern ideas of infection and immunity are incorporated and the vaccine theories are given a statement. worthy of the present confidence given them. It is a good book for the practitioners library and we have no doubt it will continue the favorite text manual for students.

Dental Materia Medica, Therapeutics and Prescription Writing. By Eli H. Long, M.D., Professor of Materia Medica and Therapeutics, Medical and Dental Departments, University of Buffalo, New York. New (3d) edition, thoroughly revised. Octavo, 311 pages, with 6 engravings and 18 colored plates. Cloth, $2.75, *net.* Lea & Febiger, Philadelphia and New York, 1909.

The third edition of this work is before us. It is not often that a medical man has succeeded in producing a text book that has become popular with dental teachers. Professor Long has done a remarkable piece of work which shows the genius of the man, in gaining such an appreciation of the needs of the dental practitioner as have enabled him to put his knowledge into a book and adopt it to the needs of the dental therapeutist. The book has gained a deserved popularity, largely because it was the first book on dental materia medica that had a scientific basis, with a practical application. It is adequately comprehensive and contains no useless padding. The present edition retains all the good points of the former editions, and the editions to the chapter on anesthetics brings it up to date on this important subject. We predict for this book the continued confidence and favor of dental teachers and students.

